# Genetic Renal Diseases: The Emerging Role of Zebrafish Models

**DOI:** 10.3390/cells7090130

**Published:** 2018-09-01

**Authors:** Mohamed A. Elmonem, Sante Princiero Berlingerio, Lambertus P. van den Heuvel, Peter A. de Witte, Martin Lowe, Elena N. Levtchenko

**Affiliations:** 1 Department of Pediatric Nephrology & Development and Regeneration, University Hospitals Leuven, KU Leuven—University of Leuven, Herestraat 49, Box 817, 3000 Leuven, Belgium; mohamed.abdelmonem@kasralainy.edu.eg (M.A.E.); santeprinciero.berlingerio@kuleuven.be (S.P.B.); bert.vandenheuvel@med.kuleuven.be (L.P.v.d.H.); 2 Department of Clinical and Chemical Pathology, Faculty of Medicine, Cairo University, 11628 Cairo, Egypt; 3 Department of Pediatric Nephrology, Radboud University Medical Center, 6525 GA Nijmegen, The Netherlands; 4 Laboratory for Molecular Bio-Discovery, Department of Pharmaceutical and Pharmacological Sciences, KU Leuven—University of Leuven, 3000 Leuven, Belgium; peter.dewitte@kuleuven.be; 5 Faculty of Biology, Medicine and Health, University of Manchester, Manchester M13 9PL, UK; martin.p.lowe@manchester.ac.uk

**Keywords:** pronephros, zebrafish, genetic renal diseases, CRISPR, morpholino, pathophysiology, new therapies

## Abstract

The structural and functional similarity of the larval zebrafish pronephros to the human nephron, together with the recent development of easier and more precise techniques to manipulate the zebrafish genome have motivated many researchers to model human renal diseases in the zebrafish. Over the last few years, great advances have been made, not only in the modeling techniques of genetic diseases in the zebrafish, but also in how to validate and exploit these models, crossing the bridge towards more informative explanations of disease pathophysiology and better designed therapeutic interventions in a cost-effective in vivo system. Here, we review the significant progress in these areas giving special attention to the renal phenotype evaluation techniques. We further discuss the future applications of such models, particularly their role in revealing new genetic diseases of the kidney and their potential use in personalized medicine.

## 1. Introduction

The zebrafish (*Danio rerio*) has gained much attention over the last few years. Slowly and steadily it has become a highly successful lower vertebrate animal model to study developmental genetics and disease pathophysiology and served as an in vivo system for the trial of novel therapeutic agents, thus bridging the gap that previously separated invertebrates and mammals in animal research [1,2,3]. Zebrafish models of human disease retain many of the advantages of mammalian models and at the same time overcome many of their limitations. Anatomically and histologically, zebrafish have retained most of the mammalian organs, tissues, and cellular systems together with their associated physiological functions. Furthermore, they have rapid ex utero development, transparent fertilized embryos, much higher fecundity at a fraction of the maintenance cost of mammalian models, and most importantly, a well-studied genome with the availability and ease of gene editing technologies [1,4,5,6].

**Figure 1:**
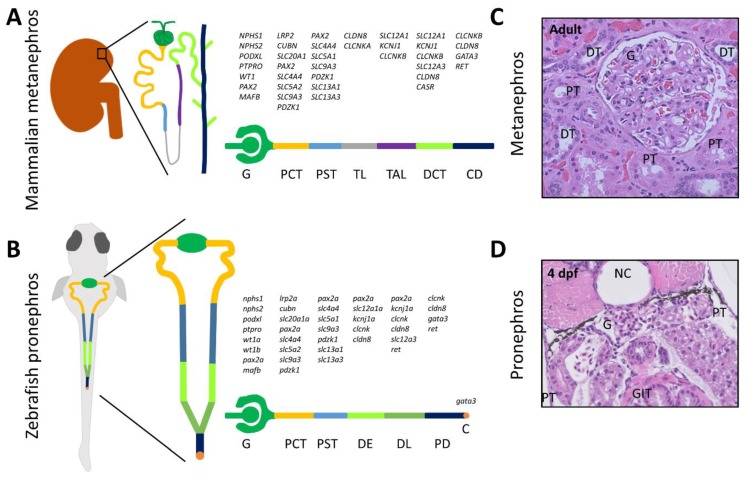
Anatomy, patterning, and histology of the mammalian adult nephron and zebrafish larval pronephros. The segmented nephron distribution of genes expressed in the mammalian nephron (**A**) and zebrafish pronephros at 48 h post fertilization (hpf) (**B**), shows major similarities between different segments of both nephrons [7,8,9,10,11,12]. All gene symbols are in accordance with the Hugo Gene Nomenclature Committee (HGNC) guidelines. Hematoxylin and eosin stained images of cut sections of the human metanephros (**C**) and zebrafish pronephros at the level of the glomerulus and proximal tubules in 4 days post fertilization (dpf) larvae (**D**) showing basic similar architecture. Abbreviations: C, cloaca; CD, collecting duct; DCT, distal convoluted tubule; DE, distal early tubule; DL, distal late tubule; DT, distal tubule; G, glomerulus; GIT, gastrointestinal tract; NC, notochord; PCT, proximal convoluted tubule; PD, pronephric duct; PST, proximal straight tubule; PT, proximal tubule; TAL, thick ascending limb of Henle; TL, thin limb of Henle.

The zebrafish embryonic kidney (pronephros) is of particular interest to researchers. It consists of a pair of segmented pronephric tubules sharing a fused glomerulus and showing remarkable histological and functional similarities to the mammalian adult nephron (Figure 1).

The pronephros is formed at 24 h post fertilization (hpf), and starts blood filtration at approximately 48 hpf [12]. At 10 days post fertilization (dpf), in order to cope with the increased osmoregulatory demands of the growing juvenile fish, mesonephric nephrons start forming from cell clusters of nephron progenitors embedded in stroma composed of hematopoietic tissue and expressing *wt1b*, *pax2a*, and *lhx1a* at the caudal end of the swim bladder. These mesonephrons fuse with the distal pronephric tubules to eventually form the mesonephric kidney, which remains during the whole adult life of the zebrafish [7]. This differs from mammals, which develop the ureteric bud from the nephric duct during embryonic life (at 5th week of gestation in humans), giving rise to the final metanephric kidney [13]. Another major difference in nephron structure between zebrafish and mammals is the absence of a loop of Henle in zebrafish, which acts in mammals as a countercurrent multiplier to produce the medullary osmotic gradient essential for water reabsorption [14].

Although adult zebrafish models are more suited to certain types of studies, especially involving the endocrine function of the kidney or the regenerative capacity of the adult zebrafish kidney [15,16,17], zebrafish embryos and larvae are by far more commonly used to model genetic renal diseases. This is mainly due to the large number of embryos and larvae that can be generated and studied per mating, and also due to the anatomical simplicity and histological and functional similarity of the larval pronephros to the human nephron [12].

In the current review, we present the basic concepts behind the modeling of genetic renal diseases in the zebrafish, outlining the advantages but also some of the limitations. We also discuss the techniques available for functional analysis of the pronephros and the potential future applications of such genetic models.

## 2. Methods for Genetic Modeling

Although zebrafish models have been commonly used for the investigation of genetic abnormalities implicated in human disease since the mid-1990s [18,19], the earliest attempt of a whole zebrafish genome sequence was first made public by the Sanger Institute, UK in 2002 and the completed reference genome was reported in 2013 [4]. The latest version of the zebrafish genome (GRCz11) was released in May 2017 by the Genome Reference Consortium (http://genomereference.org). Among 26,000 predicted zebrafish protein coding genes, over 18,000 genes (69%) have an orthologue in the human genome. These include over 2600 genes with a human orthologue known to cause disease, constituting over 80% of the total genes linked to disease in humans [4].

In general, there are two main approaches for studying the function of a gene in vivo; forward genetics and reverse genetics. Table 1 provides a comparison of the commonly used methodologies for assessing gene function and performing disease modeling in the zebrafish. Forward genetic screening was the initial approach to identify genes associated with phenotypic changes, including those seen in disease. The method involves inducing random DNA mutations in germ cells of adult males through gamma irradiation [20] or more commonly by using chemical mutagens, such as *N*-ethyl-*N*-nitrosourea (ENU) [21]. This is usually followed by mating with wild-type females, propagating their offspring through inbreeding to obtain homozygous mutants, isolating offspring with the phenotype of interest, then identifying the mutated genes through positional cloning, linkage mapping, or whole exome and whole genome sequencing [22]. Another method of forward genetic screening is insertional mutagenesis, during which transposable DNA elements (transposons) or more commonly retroviral vectors are injected in late blastulae stage of zebrafish development (512–2048 cell embryos) [23]. These vectors insert foreign DNA randomly at different locations of the zebrafish genome; however, the mutagenic rate is only about 10% of that of ENU mutagenesis [24]. During screening, foreign DNA sequence can be used as a tag to identify the mutated genes, which is far easier than the screening techniques developed for chemical mutagenesis [25].

In reverse genetics, the approach is to first identify genes of interest, and then target them specifically either by knocking-down expression, editing the gene to create knock-out or knock-in alleles, or in some cases over-expressing the gene product, followed by evaluation of the phenotype [28]. Among reverse genetics techniques, two techniques stand out. The morpholino (MO) antisense oligonucleotide approach due to its simplicity and lower cost and the clustered regularly interspaced short palindromic repeats (CRISPR)/Cas9 system due to its high specificity and efficiency and the permanent genetic model obtained [6].

### 2.1. Morpholino Antisense Oligonucleotides

**Figure 2:**
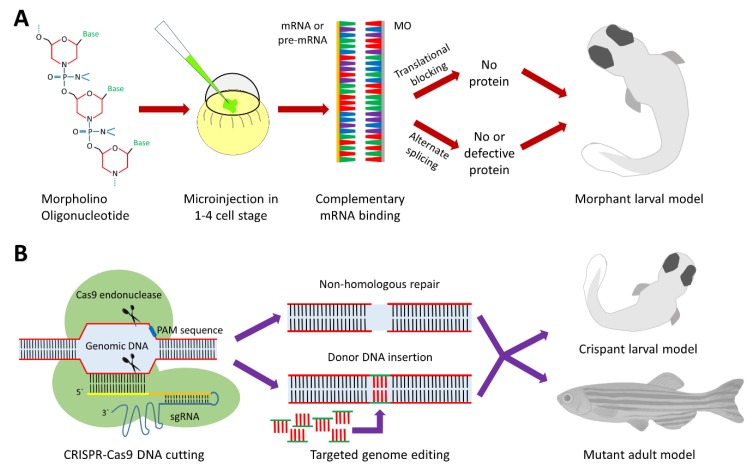
Reverse genetics in zebrafish using morpholinos and CRISPR-Cas9. (**A**) Morpholino antisense oligonucleotides (MOs): Morpholinos are synthetic single stranded nucleic acid analogues with a methylenemorpholine ring backbone replacing the sugars normally present in nucleic acids. The designed MO is injected at the 1-4 cell stage embryo, binds specifically to its target mRNA or pre-mRNA. Depending on whether the MO binds to the translation start site or a splice donor or acceptor site, it will either block protein translation or cause alternate splicing to produce a defective message that is either degraded, resulting in loss of protein expression, or still present in which case it will produce a defective protein. The resulting phenotype typically lasts for a few days. (**B**) Clustered regularly interspaced short palindromic repeats (CRISPR)/Cas9: The bacterial endonuclease enzyme is a large protein encoded by the *cas9* gene. Specificity of the DNA strand cleavage is dependent on the pairing between the single guide RNA (spacer domain) and the complementary DNA target (protospacer domain). The Cas9 protein has also a domain that binds to a short sequence of target DNA, named the protospacer adjacent motif (PAM), which is found directly downstream of the target sequence in the genomic DNA, on the non-target strand. Because the spacer domain sequence provides at least 20 nucleotides of specificity in addition to the specificity of the PAM sequence, the CRISPR-Cas9 system can uniquely cleave DNA at a highly specific target site [6,34]. The cleaved DNA is then left to the non-homologous end-joining repair machinery, which can result in random deletions or insertions and loss of a functional allele. Alternatively, if a synthesized DNA template is introduced, homology-directed repair results in the generation of an engineered mutant allele at the break site [35].

MO are synthetic single stranded analogues of nucleic acids. They are usually injected into one to four cell stage zebrafish embryos, and by binding to the complementary mRNA molecule, they can either block the translation of a target gene, or disrupt splicing (if they bind to a region including a splicing donor or acceptor site) [26]. Because MO are resistant to degradation by nucleases, their gene silencing effects are very efficient. However, due to the proliferation of cells in the growing embryo which results in dilution of the MO in morphant larvae, the effect of suppression is gradually lost over time such that down-regulation typically only lasts for up to a few days [29] (Figure 2A). A potential advantage of MOs is that because they acutely down regulate genes, they may produce more severe phenotypes when compared to stable genetic knock-out models in which there is the possibility of compensatory or adaptive responses [30]. The main disadvantage of MOs is their potential to produce off-target genetic effects, most importantly the non-specific activation of the pro-apoptotic p53 pathway. The simultaneous use of an anti-p53 MO is an important control measure to overcome this effect [31]. Even then, it is always important to control for off-target effects when using MO. Another major concern, because of the transient nature of MOs, is the reproducibility of phenotypic effects, thus the standardization of injection protocols needs to be emphasized [32]. The disease models solely based on MO knockdown need to be validated in corresponding genetic models; however, MOs remain a valuable tool for investigating gene function in zebrafish [32,33].

### 2.2. CRISPR-Cas9

On the other hand, the recent CRISPR-Cas9 technology provides a mostly permanent and very specific type of genetic manipulation. Cas9 is one of many RNA guided endonuclease enzymes derived from the immune system of bacteria and archaea for natural defense against invading viruses [36]. Over the past few years, the CRISPR-Cas9 system has been adapted successfully for use in editing the genomes of a wide variety of multicellular and complex organisms, including zebrafish, mice, and humans [27,37,38,39,40,41,42]. Cas9 is attached to two RNA guide molecules: the trans-activating CRISPR RNA (tracrRNA) and the CRISPR RNA (crRNA) to form a trimeric complex in bacteria named the Cas9 holoendonuclease system. In an experimental setup, a specifically designed single guide RNA (sgRNA) usually replaces the tracrRNA-crRNA complex [35] (Figure 2B). CRISPR-Cas9 technology can be used to produce transient knockdown larval models (crispants) [43], which are similar to morphant zebrafish larvae in many aspects but lacking the non-specific toxicity of MOs [32]. However, CRISPR technology is more commonly used to grow fish to adulthood and produce permanent genetic zebrafish models.

The advantages of CRISPR technology include its high efficiency, specificity and affordability, the possibility of both knock-out and knock-in models, and the potential to study the phenotypes associated with specific human mutations through generating the same mutations in the zebrafish. Other techniques for genome editing include zinc finger nucleases (ZFNs) and transcription activator-like effector nucleases (TALENs). However, both systems are less tractable than CRISPR-Cas9, which uses a universal targeting mechanism [6,44]. Furthermore, CRISPR-Cas9 is far more efficient than ZFNs and TALENs in achieving targeted mutagenesis in the zebrafish [45]. Recently, the CRISPR-Cas13 system was adapted for both RNA knockdown and RNA editing in human cells [46]. The system is extremely precise and has many potential applications including splicing modifications, targeted localization of transcripts, epitranscriptomic modifications, and the ability to correct certain disease relevant mutations at the RNA level. Another recent alternative adaptation to the traditional CRISPR-Cas9 approach is the engineered Cas9-cytidine deaminase fusion, which was recently implemented in human cells [47]. This technique is capable of substituting single base pairs with high efficiency by using a specifically designed inactive Cas9 protein coupled with a cytidine deaminase enzyme and an inhibitor of base excision repair. Although these systems are yet to be tried in zebrafish, they can definitely expand the toolkit for genome editing.

## 3. Assessment of the Renal Phenotype

A number of histopathological lesions seen in diseases affecting the mammalian kidney can be recapitulated in the zebrafish [12,48,49]. Nevertheless, for the larval zebrafish to be a valid model to study renal disease and potential new therapies, the availability of methods for the assessment of renal function in this organism is necessary. The evaluation of renal function in murine models is not much different from humans. In mice, blood and urine samples can be easily obtained to measure various aspects of renal function, such as serum creatinine levels and urinary protein/creatinine ratios to evaluate glomerular function, and serum electrolytes and urinary low molecular weight proteins and other solutes concentrations to evaluate renal tubular function. However, in zebrafish larvae these methods are not currently feasible. A new panel of methodologies therefore had to be developed to accurately evaluate different aspects of renal function in the larval zebrafish.

### 3.1. Evaluation of Zebrafish Survival, Development, and Morphology

Because of the available numbers, zebrafish embryos are extremely useful for the accurate evaluation of the phenotypic picture based on survival, development, and morphological characteristics in genetic disease models. This is particularly important in genetic renal diseases as many of them are characterized by increased mortality rates, delayed development, or morphological aberrations in the zebrafish [12,48,50,51]. The zebrafish pronephros becomes functionally active at 40–48 hpf [12]. Thus, depending upon the gene involved, impairment of renal function can result in systemic phenotypes at early stages, which can be seen as fluid retention and edema, which ultimately can affect viability [12]. Hence, survival and developmental and morphological changes can be important in determining the systemic effects of gene disruption. Common morphological defects seen upon severe renal impairment include pericardial edema and total body edema although such phenotypes are not exclusive to renal disorders. Other body deformities, such as hydrocephalus, microphthalmia, curved body, and left-right axis asymmetry are more frequently associated with ciliopathies, which often also result in renal cysts [52,53,54].

### 3.2. Evaluation of Glomerular Function

The filtration of various molecules has been used to assess the functionality of the glomerular filtration barrier in the zebrafish. Of particular importance are the dextran based compounds, as they are very commonly used for this purpose [12]. Dextran is a complex polysaccharide formed of branched glucose moieties. Dextran has many advantages as a measure of the integrity of the glomerulus including its variable size, as it can be obtained between 3 and 2000 kilodaltons (kDa). Furthermore, it is inert, with no induced immune reaction when given intravenously, and it can be labelled with fluorescent tags for visual detection in the vasculature and tissues of the transparent zebrafish larva [12,55].

**Figure 3:**
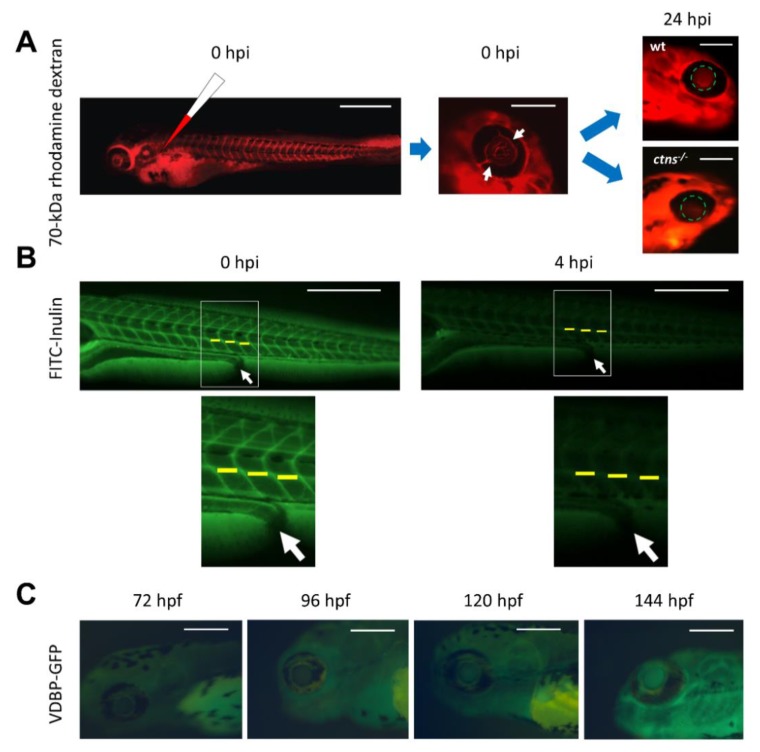
Evaluation of glomerular function in the zebrafish. (**A**) 70-kDa rhodamine labelled dextran is injected in zebrafish larvae at 72 hpf (hours post fertilization). Immediately after injection (0 hpi, hours post injection), the success of intravascular injection is confirmed through observing the fluorescent dye in all capillaries including those situated in the retinal vascular bed (white arrows). At 24 hpi, the fluorescence signal intensity is quantified in fixed diameter circles in the retinal vascular bed using image-processing software, such as ImageJ. In wild type larvae, glomerular function is preserved and fluorescence accumulates in the retinal vascular bed as expected, while in the cystinosis mutant (*ctns*^−/−^) larvae, the glomerular barrier is defective [48] and the 70-kDa dextran is lost in urine, thus the fluorescence intensity is significantly reduced (bars from left to right = 500 µm, 200 µm, and 200 µm). (**B**) FITC labelled inulin is injected at 96 hpf. Initial images are obtained immediately after injection (0 hpi) and 4 h later (4 hpi). The intensity of fluorescence is quantified over the cardinal vein at the 14th, 15th, and 16th somites (yellow lines). The average is determined for each fish and for each time point, then glomerular filtration rate (GFR) is expressed as the percentage decline of fluorescence over the 4 h incubation period (bars = 500 µm), white arrows refer to the site of the cloaca. (**C**) The VDBP-GFP transgenic zebrafish line at 72, 96, 120, and 144 hpf. The fluorescence intensity naturally accumulates in the retinal vascular bed over time with the increased production of the vitamin D binding protein (bars = 200 µm).

Below 10 kDa, dextran is promptly filtered by the glomerulus, whereas at higher molecular weights the filtration is less efficient (70 kDa) or does not occur at all (500 kDa) [56]. Thus, both the glomerular filtration rate and the integrity of the glomerular barrier can be evaluated using the injection of low molecular weight (3–10 kDa) and high molecular weight (70–500 kDa) fluorescent dextran, respectively [12,57,58,59,60]. Furthermore, both tracers can be simultaneously evaluated using different fluorophores [61]. The main advantage of such a technique is the ability to perform live imaging of fish larvae at different time points to evaluate fluorescence intensity loss in the retinal vascular bed [62] (Figure 3A), the heart [57], or over a major vessel, such as the cardinal vein, as a readout of clearance by glomerular filtration [61]. Of note, the size selectivity of the glomerular barrier is not well established in the zebrafish during the first 3 dpf [55], so it is important to test for glomerular proteinuria starting from 4 dpf. Another way to evaluate clearance is through the evaluation of fluorescence intensity in fixed sections at the tubular level, which allows for the simultaneous evaluation of glomerular and tubular functions [48,55,56].

Another polysaccharide that can be used for the evaluation of glomerular function in the zebrafish is inulin (Figure 3B). Inulin clearance measured after the intravascular injection of FITC-inulin is a good alternative to dextran in determining the glomerular filtration rate, especially because inulin is freely passing through the glomerular barrier and not reabsorbed or secreted from the proximal tubules, making it an ideal molecule to assess the glomerular filtration rate [63,64]. Inulin clearance is the current gold standard to assess the glomerular filtration rate (GFR) in humans [65]; however for this purpose, it has to be measured in the plasma and urine of patients.

A major drawback for the practical application of such techniques is the need for injection of the fluorescently tagged reporter into the vasculature, which is a labor intensive and time-consuming procedure, especially when applied to large numbers of larvae. New transgenic zebrafish lines expressing fluorescently-tagged plasma proteins have been developed to overcome such a hurdle [66,67,68]. In humans, the most commonly used plasma protein to evaluate glomerular permeability is albumin, as it constitutes approximately 50% of the total plasma protein, which is why the assessment of the urinary albumin/creatinine ratio is a common practice for the evaluation of the glomerular barrier integrity of the human kidney. However, a gene encoding albumin is absent from the zebrafish genome [69]. The likely zebrafish equivalent of albumin is vitamin D binding protein (VDBP), which belongs to the same family of carrier proteins as albumin, and, like albumin, is produced in the liver and secreted in the bloodstream [70]. When fused to GFP, VDBP has a molecular weight and electric charge approximate to that for human albumin (79.6 kDa vs. 66.5 kDa, and an isoelectric point 5.97 vs. 5.67, for VDBP-GFP vs. human albumin, respectively), so they should behave in a similar way at the glomerular filtration barrier [68]. In a transgenic zebrafish line expressing VDBP-GFP, the integrity of the glomerular barrier can be evaluated in a very similar way to that for high molecular weight fluorescent dextran by assessing fluorescence in the retinal vascular bed (Figure 3C), the cardinal vein or over the heart, or in the case of a defective glomerular barrier, in the proximal tubules [64]. Recently, 4D in vivo imaging using two-photon microscopy allowed for the simultaneous assessment of fluorescence intensity of the VDBP-GFP fusion protein in the vasculature and proximal tubules of live zebrafish larvae, which gives the opportunity for dynamic monitoring of the glomerular filtration barrier [71].

A transgenic zebrafish line co-expressing VDBP-GFP from the liver and a nitroreductase enzyme within podocytes has also been generated [68]. Due to the ability of nitroreductase to convert metronidazole to a cytotoxin, this transgenic line allows for the inducible and acute damage of podocytes and the analysis of glomerular integrity following such treatment. It may also be used to study podocyte regeneration following metronidazole washout [12].

### 3.3. Evaluation of Tubular Function

#### 3.3.1. Tubular Endocytosis

Receptor mediated endocytosis by proximal tubular epithelial cells (PTECs) is an important process by which the kidney can minimize the urinary losses of important proteins, vitamins, hormones, and other solutes through their uptake from the tubular lumen. Megalin and cubilin are major multi-ligand transmembrane receptors that are mainly expressed at the luminal brush border of PTECs and are largely responsible for this endocytic uptake [72]. Loss of megalin in humans causes Donnai–Barrow syndrome, which is characterized by low molecular weight proteinuria amongst other symptoms [73]. Both receptors are highly evolutionary conserved between different species, and the zebrafish is no exception [74].

**Figure 4:**
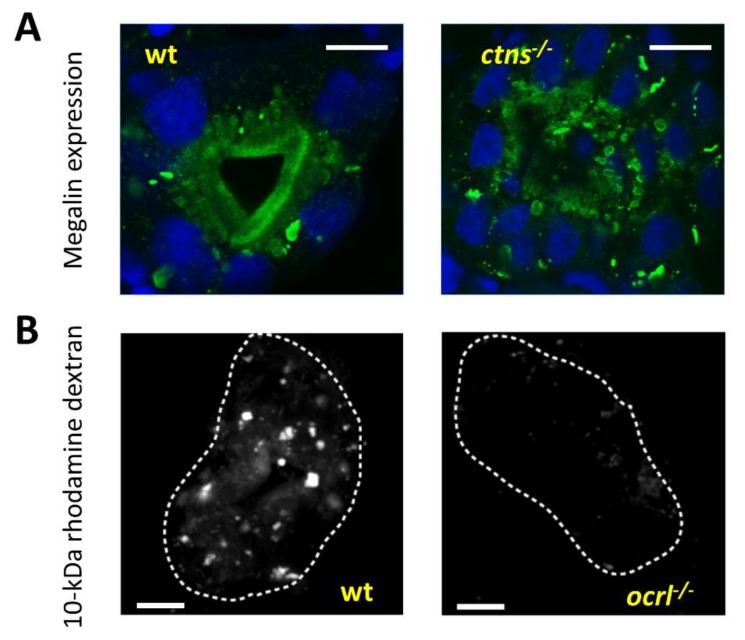
Evaluation of proximal tubular endocytosis. (**A**) Evaluation of megalin localization: Transverse confocal fluorescence images of the proximal pronephric region of wild type (wt) and cystinosis mutant larvae (5 dpf) showing endogenous megalin distribution with an anti-megalin antibody. In the wild type zebrafish, megalin is localized predominantly at the luminal brush border of the pronephric tubules, while in the cystinosis zebrafish, megalin abundance is significantly reduced in the brush border and it is mainly trapped in multiple subapical and cytoplasmic vacuoles, demonstrating defective endosomal trafficking in the cystinosis zebrafish (bars = 5 µm). (**B**) Transverse fluorescent images of the proximal pronephric region in wt and *ocrl* mutant zebrafish larvae after 2.5 h of 10-kDa Alexa488-conjugated dextran injection at 72 hpf. In wild type dextran is normally reabsorbed at the proximal tubular level, while in the Lowe syndrome model dextran reabsorption is almost completely absent (bars = 5 µm). White dashed lines represent the outline of the proximal tubule.

Megalin, encoded by *lrp2a* gene, is important for proximal tubular function in the zebrafish [75]. Loss of megalin protein in zebrafish (*bugeye* mutant), or its depletion induced by *lrp2a* MO, abrogates endocytosis and results in loss of apical endosomes in the proximal pronephric duct epithelium [75,76]. This is similar to what is seen in megalin knockout mice [77], indicating the conservation of the megalin retrieval pathway between the larval zebrafish pronephros and the mammalian kidney. Many proximal tubular diseases modeled in zebrafish alter megalin expression and function resulting in defective tubular reabsorption similar to the *lrp2a* mutant, such as observed in the cystinosis *(ctns)* and Lowe syndrome *(ocrl)* models [48,49] (Figure 4A,B). A good way to monitor endocytosis in the pronephros is performed through using low molecular weight fluorescent dextran (10 kDa or less). This fluid phase tracer is efficiently filtered and taken up by endocytosis into the pronephros [75]. Another tracer that can be used to more directly assess megalin-dependent endocytosis is fluorescently conjugated receptor-associated protein (RAP), which is a physiologic chaperon for megalin [78]. Loss of megalin abrogates endocytosis of both tracers.

Similarly, plasma proteins such as VDBP, can also be reabsorbed by megalin-dependent endocytosis upon disruption of the glomerular filtration barrier [68,79]. The quantitation of the fluorescence signal of different tagged molecules over the pronephric tubules, especially around the brush border is a very good way of testing the efficiency of the PTECs endocytic machinery, provided that proper control groups are used. Recently, a fluorescent low molecular weight probe (PT-yellow) has been developed that is selectively taken up into the zebrafish proximal tubules simply by soaking larvae in the compound, with no need for injection [80]. This non-toxic molecule accumulates in endocytic organelles, but whether the mechanism of uptake is endocytosis dependent, remains to be determined. Interestingly, the strength of PT-yellow accumulation was significantly reduced upon exposure to gentamicin [80], which has been shown previously to ablate PTECs in the zebrafish [62]. Several transgenic zebrafish lines have been developed to mark the proximal tubules with fluorescent reporters. Some of these reporters are expressed only in the proximal part of pronephric tubule, such as those for *gtshβ* [81] and the *tg(PT:EGFP)* transgenic line, which was isolated serendipitously during the generation of *sox10:EGFP* fish [82], while other reporters mark the entire pronephric tubules, such as *enpep* [83].

#### 3.3.2. Ion and Small Solute Transport

Zebrafish are hyperionic and hyperosmotic in comparison to their aquatic environment. This results in the passive loss of ions and uptake of water along their electrochemical and osmotic gradients, respectively [84]. To maintain physiological balance, compensatory transport systems to reabsorb ions and control water balance must exist. The cells responsible for maintaining this delicate balance are specialized ionocytes that are mainly located in the skin of embryos/larvae and gills of adult zebrafish. However, both pronephric and mesonephric renal tubular cells also express many of the ion channels present in the skin or gills, and together, the kidney and skin/gills of zebrafish work cooperatively to regulate the balance of different electrolytes [85]. Similarly, the transport of small molecules such as glucose also occurs in the kidney as the major zebrafish glucose transporter (slc2a2), which is an orthologue of the human glucose transporter (GLUT2), is expressed in the zebrafish pronephros [86]. Few studies have tested ion homeostasis in the zebrafish. A potential functional assay challenges zebrafish embryos with water supplemented with different concentrations of ions to monitor the physiological response of zebrafish to changes in ionic composition of the environment. This is usually followed by the quantitation of target ions in larval homogenates. Recently, the importance of *casr* and *arl15b* genes for the maintenance of calcium [87] and magnesium [88] homeostasis, respectively, was reported using this evaluation method.

### 3.4. Evaluation of Renal Cysts

Forward genetic screens in the zebrafish confirmed the connection between pathogenic mutations in genes controlling the formation and function of cilia and the development of cystic kidney diseases [50,89]. The proper visualization of renal cysts early during the first few days of zebrafish embryonic development is essential to categorize the disease phenotype and to evaluate the response to potential therapy. Although it is relatively easy to visualize renal cysts in the transparent larvae simply by monitoring the pronephros using light microscopy, detecting smaller cysts or monitoring the rate of cyst development might pose a challenge. A transgenic line *Tg (wt1b::GFP)*, showing fluorescence associated with the Wilms tumor 1b protein, which is mainly expressed in the glomerulus and proximal tubules of the developing embryo [90], can facilitate the identification and monitoring of small renal cysts in vivo [51,91,92]. The model can be also used to test new therapeutic approaches, and their effects upon cyst formation [93]. Another transgenic zebrafish line *Tg (Arl13b::GFP)* marks the ciliary membrane and thus can facilitate the study of tubular cilia morphology and abundance [94].

## 4. Characterized Zebrafish Models of Genetic Renal Diseases

The number of zebrafish models generated to study genetic renal diseases has grown exponentially over the last decade. Table 2 provides a list of the main characterized embryonic and larval models of genetic renal diseases in the zebrafish, with phenotypic features and the methods used to create the models. The majority of zebrafish models are created by MO injection, thus validation in permanent mutant genetic models is still needed for most disorders. The main disease categories studied in zebrafish are genetic glomerular and tubular disorders, renal ciliopathies, and congenital anomalies of the kidney and urinary tract (CAKUT).

In most cases, disease phenotypes appear to be recapitulated in the zebrafish, although this is not true in all cases. For instance, in an Alport syndrome model due to loss of *col4a5* (*dragnet* mutant), only ocular but no glomerular defects have been observed [95]. Another example is the Branchio-oto-renal syndrome caused by *EYA1* gene deficiency. Both craniofacial and ear malformations were evident in the *eya1* MO zebrafish model similar to those of the human disease. However, abnormal renal development was not observed because the gene is expressed relatively late at the mammalian metanephric stage, and is completely absent during early renal development in the zebrafish [96].

Although, human genes responsible for hereditary nephrolithiasis syndromes, such as cystinuria (*SLC3A1*, *SLC7A9*), primary hyperoxaluria (*AGXT*, *GRHPR*, *HOGA1*), Dent’s disease (*CLCN5*), and xanthinuria (*XDH*), have counterpart genes in the zebrafish genome, and some of them are reported to be expressed in the zebrafish pronephric tubules [97,98], no zebrafish models have been created for these disorders. This is probably due to the different physiological aspects concerning urine formation in the zebrafish, particularly their lack of need to concentrate urine in the fresh water environment [14]. However, it is worth noting that adult zebrafish are capable of developing kidney stones as evident by the mutant model for *trpm7* gene, which codes for a transient receptor potential cation channel, that is expressed in the mesonephric tubules [99].

## 5. Drug Discovery and Validation

The rapid development of zebrafish allows drug screening to be performed at embryonic and larval stages, prior to the stage at which the animals become protected by ethical regulations, which is normally at 6 days post fertilization in most countries [154]. Furthermore, their abundance, small size, ease of handling, transparency, low cost, and most importantly, the availability of clear phenotypic assays, makes zebrafish an extremely powerful model organism for in vivo therapeutic drug screening and discovery [2]. Furthermore, major drug classes affecting human cell physiology, such as prostaglandins, hematopoietic factors, and drugs affecting glucose homeostasis, perform in the zebrafish in a very similar manner to humans [155,156,157]. Since the pioneering work of Cao et al., through chemical modifier drug screens to unravel the beneficiary effects of histone deacetylase inhibitors in the treatment of polycystic kidney disease zebrafish models [158], multiple studies have tested novel therapeutic agents in renal zebrafish models [93,130,159,160].

Recently, PI3-kinase inhibitors were reported to rescue the cellular, phenotypic, and renal functional defects of the Joubert syndrome associated *inpp5e* mutant zebrafish larvae by decreasing 3-phosphoinositide levels [130]. On the other hand, *nek8* mutants, associated with syndromic renal cystic dysplasia showed increased signaling of the transcriptional factor YAP, which is involved in the Hippo signaling pathway controlling organ size, cell proliferation, and apoptosis. The treatment with verteporfin, an inhibitor of YAP transcriptional activity, partially rescued the abnormalities seen in zebrafish mutant embryos [159].

Zebrafish is also an excellent in vivo model to study drug toxicity and drug–drug interactions, especially since zebrafish larvae at 72 hpf have a fully functional liver, expressing 94 different Cytochrome P450 enzyme genes, many of them having human orthologues [161]. Moreover, zebrafish can be used for the high throughput drug–drug interaction screening for diseases requiring multiple drug therapy [162].

## 6. Limitations of Zebrafish Models

In spite of the many advantages of zebrafish, they still have some limitations that must be taken into account when considering generating a disease model. General limitations applying to zebrafish models include the presence of many duplicated genes, caused by a whole genome duplication event in early teleost evolution 320–350 million years ago, followed by the retention of many duplicated genes in different species [163]. Due to this phenomenon, many mammalian protein coding genes (over 3000 mammalian genes), have two or more zebrafish counterparts, which may code for similar proteins with similar functions [4]. In such cases, biallelic mutations in a single gene may not be enough to produce the desired phenotype. Furthermore, inbreeding of mutants can encourage adaptive or compensatory responses [30].

As mentioned previously, some larval zebrafish models do not recapitulate the expected disease phenotype, which could be due to functional redundancy with duplicated paralogues, or other genes from the same family, or the lack of expression of the gene of interest at the larval stage. Also, zebrafish models are probably unsuitable to study genetic diseases affecting water homeostasis, such as hereditary nephrogenic diabetes insipidus, which is caused by either *AVPR2* or *AQP2* defects in humans [164]. A true orthologue of *AQP2* (aquaporin 2) is lacking in the zebrafish [165]. This could be due to the different aquatic environment and different controlling mechanisms of water and ionic balance in the zebrafish [84]. Furthermore, some genes may be completely lacking in the zebrafish, such as the ENaC (epithelial Na channel) subunits (*SCNN1A*, *SCNN1B*, *SCNN1G*, and *SCNN1D*) [166], mutations in which cause Liddle syndrome and pseudohypoaldosteronism type 1 in humans.

Although there appears to be a high degree of conservation between zebrafish and mammals regarding drug sensitivity and toxicity, it is always difficult to correlate therapeutic doses in zebrafish to mammalian doses, especially when considering that zebrafish physiology, mode of drug administration, and hemodynamics are different. Furthermore, the exact amount of the drug taken up by the larvae is usually hard to determine [167]. Another general disadvantage concerning drug therapy in zebrafish models is the lack of a complete picture of drug handling by certain organs, such as the lungs and mammary glands, as they are absent in the zebrafish [168].

## 7. Future Perspectives

Zebrafish models have been advancing our knowledge of renal development and disease at an ever-increasing rate. One of the most important research targets of zebrafish is to understand the functions of genes involved in renal regeneration. Zebrafish has significantly lower number of nephrons in their adult mesonephric kidney (150–300) [67], compared to approximately 12,000 nephrons in the mouse and one million nephrons in the human adult metanephros [11]. However, zebrafish retain the ability to add new nephrons to their mesonephric kidney in the juvenile and adult stages in a process called neonephrogenesis [17,67,169]. In this respect, zebrafish genetic models can help understanding the role of key mechanisms of these processes, which may guide the discovery of similar mechanisms that are dormant and can be rejuvenated in mammals upon renal injury.

Although many genes have been identified recently, in the era of wide scale whole exome and whole genome sequencing, as the cause of monogenic hereditary renal disorders [151,170,171,172], it is conceivable that many genes affecting the kidney are still waiting to be attributed to human syndromes. Zebrafish could be uniquely useful in this regard. Through the ease of both genetic manipulation and renal phenotypic validation, novel genes identified in the zebrafish can be linked to a renal phenotype, and if the gene is sufficiently conserved between zebrafish and mammals, it is highly likely to be attributed later to a human disease [173,174]. Furthermore, the confirmation of such potential genetic candidates can be investigated by comparing the ability of injected wild type human gene mRNA, or mRNA with a pathogenic human mutation (e.g., a single nucleotide polymorphism) to rescue the zebrafish phenotype [175].

Another future application of zebrafish research is its potential for developing strategies for personalized medicine. Patients of a single monogenic disease vary in both clinical severity and response to therapeutic interventions. This could be explained by either the variability of the causative mutations or the presence of unknown genetic modifiers within the patient genome [176]. Due to the recent availability of extensive sequencing techniques, it has become easier than ever to identify associated mutations in potential modifier genes, while diagnosing the causative mutations in the responsible gene. The availability of reverse gene editing techniques, such as CRISPR-Cas9, that can introduce specific mutations in the zebrafish genome, makes studies of personalized medicine much more feasible. There are hurdles yet to overcome in this regard, especially the still reported off-target effects of CRISPR, which can be up to five mismatched bases in the recognized sequence [177]. However, there are multiple recent advances in the CRISPR technology aiming at minimizing its off-target effects without losing much of its on-target efficiency. These include the structure-guided protein engineering approach to create Cas9 variants that have less off-target effects [178,179], and new computational algorithms for predicting potential off-target effects for optimal design of guide RNAs [180,181,182]. Moreover, there are validated biochemical methods to screen for the off-target cleavage sites using various sequencing techniques, such as the high-throughput, genome-wide, translocation sequencing (HTGTS) [183], GUIDE-seq [184], Digenome-seq [185], and SITE-seq [186]. Although these technologies are not developed specifically for zebrafish, they and other future advances will soon allow the routine creation of specialized zebrafish models, accurately and individually mimicking human mutations in both causative and modifier genes. In addition, since zebrafish is an excellent target model for drug experimentation, this will allow the search for novel therapeutic agents to be also personalized.

## 8. Conclusions

The zebrafish has proved its significance as a valuable vertebrate model to study renal development and disease. Zebrafish are particularly amenable to genetic manipulation by novel technologies. Their phenotype in the majority of cases is faithful to the human phenotype. It is foreseeable that new genetic zebrafish models of many human hereditary renal diseases will be developed during the next few years. This, combined with better assays for evaluating renal function and the generation of new transgenic reporter lines, means that studying kidney disease and finding new therapies in these models will become even more powerful.

## Figures and Tables

**Table 1:** Attributes of key methods used to model genetic diseases in the zebrafish.

	Forward Genetics		Reverse Genetics	
	ENU Mutagenesis	Retroviral Insertion	MO	CRISPR-Cas9
Technique first described in zebrafish	Grunwald and Streisinger (1992) [21]	Lin et al. (1994) [23]	Nasevicius and Ekker (2000) [26]	Hwang et al. (2013) [27]
Genetic target	Genomic DNA	Genomic DNA	mRNA	Genomic DNA
Stage of inducing mutagenesis	Adult males	512–2048 cell stage (blastulae)	1–4 cell stage	1 cell stage
Mutation site	Random	Random	No DNA mutations	specific DNA sequence
Mutational effect	Mainly deficiency	Mainly deficiency	Deficiency	Deficiency/Gain
Difficulty of confirming the mutant genotype	Difficult	Less difficult	Easy	Easy
Efficiency of mutagenesis	Medium	Low	High	High
Mutant model	Permanent	Permanent	Transient	Permanent
Time, effort and resources	+++	++++	+	++
Off-target effects	+	+	+++	+

CRISPR, clustered regularly interspaced short palindromic repeats; ENU, *N*-ethyl-*N*-nitrosourea; MO, morpholino antisense oligonucleotides.

**Table 2:** Characterized embryonic and larval models of genetic renal diseases in zebrafish.

Disease	OMIM	Heredity	Gene	Methodology	Phenotype	Ref.
**Tubular disorders**						
Cystinosis	219800	AR	*ctns*	MO, ENU	Cystine accumulation, increased embryonic mortality, delayed development, apoptosis, defective glomerular permeability, altered tubular reabsorption, and megalin expression	[48]
Donnai–Barrow syndrome	222448	AR	*lrp2a,b*	MO, ENU	Defective endocytosis in larvae and bug eyes in adults	[75,76]
Lowe syndrome	300555	AR	*ocrl*	MO, Retroviral insertion	Increased embryonic mortality, delayed development, impaired pronephric endocytosis, altered megalin subcellular localization in proximal tubules	[49]
ADTKD	617056	AD	*sec61a1*	MO, CRISPR	Convolution defects of the pronephric tubules, pronephric tubular atrophy	[100]
Hypermanganesemia with dystonia type 1	613280	AR	*slc30a10*	CRISPR	Hypermanganesemia and fatty liver in larvae and dystonia, cirrhosis, and neurological deficits in adults	[101]
SeSAME syndrome	612780	AR	*kcnj10a*	MO	Dilated pronephric duct, pericardial edema, neurological manifestation	[102]
Proximal RTA with ocular anomalies	604278	AR	*slc4a4*	MO	Impaired renal electrolyte balance, edema, altered brain and eye development	[103]
Familial Hypocalciuric Hypercalcemia type I	145980	AD	*casr*	MO	Increased calcium content, impaired regulation of calcium metabolism	[87]
Hypomagnesemia *	------------	------------	*arl15b*	MO	Pronephric magnesium wasting, cardiovascular impairments, poorly metabolized yolk	[88]
**Glomerular disorders**						
SRNS1 (Finish type)	256300	AR	*nphs1*	MO	Ultrastructural glomerular damage, proteinuria, edema, increased embryonic mortality	[55]
SRNS2	600995	AR	*nphs2*	MO	Ultrastructural glomerular damage, proteinuria, edema, increased embryonic mortality	[55]
SRNS3	610725	AR	*plce1*	MO	Ultrastructural glomerular damage, proteinuria, edema	[104]
SRNS4	607832	AR, AD	*cd2ap*	MO	Ultrastructural glomerular damage, proteinuria, edema	[105]
Denys–Drash syndrome	194080	AD	*wt1a,b*	MO	Ultrastructural glomerular damage, proteinuria, edema, deformity, high embryonic mortality	[106]
Nail-patella syndrome	161200	AD	*lmx1b*	MO	Ultrastructural glomerular damage, proteinuria, edema	[107]
Schimke Immuno-Osseous Dysplasia	242900	AR	*smarcal1*	MO	Increased embryonic mortality, delayed development, increased apoptosis, edema, deformity	[108]
FSGS4	612551	AR	*apol1*	MO	Ultrastructural glomerular damage, proteinuria, edema	[109]
FSGS5	613237	AD	*inf2*	MO	Ultrastructural glomerular damage, proteinuria, edema	[110]
FSGS6	614131	AR	*myo1e*	MO	Pericardial edema, pronephric cysts	[111]
FSGS8	616032	AD	*anln*	MO	Ultrastructural glomerular damage, proteinuria, edema	[112]
FSGS9	616220	AR	*crb2b*	MO	Ultrastructural glomerular damage, proteinuria, edema	[113]
Von Hippel–Lindau disease	193300	AD	*vhl*	MO, ENU	Ultrastructural glomerular damage, proteinuria, edema, proximal tubular damage, increased angiogenesis	[114,115]
Glomerulopathy *	------------	------------	*shroom3*	MO	Ultrastructural glomerular damage, proteinuria, edema, gastrulation defects	[116]
Glomerulopathy *	------------	------------	*fat1*	MO	Impaired podocyte migration, glomerular defects, pronephric cysts	[92]
**Renal ciliopathies**						
ADPKD	173900	AD	*pkd1a,b*	MO, TALENs	Dorsal axis curvature in morphants and hydrocephalus, craniofacial defects, and pronephric cysts in both	[117,118]
	613095	AD	*pkd2*	MO, ENU	Dorsal axis curvature, hydrocephalus, pronephric cysts in morphants, and organ laterality defects in both	[117,119]
ARPKD	617610	AR	*dzip1l*	MO, CRISPR	Pronephric cysts, curved body, hydrocephalus, otolith defects	[51]
NPHP1	256100	AR	*nphp1*	MO	Pronephric cysts, duct dilatations, deformity	[120]
NPHP2	602088	AR	*invs*	MO	Pronephric cysts, ventral axis curvature, randomization of heart looping	[121]
NPHP3	604837	AR	*nphp3*	MO	Pronephric cysts, curved body, hydrocephalus, left right asymmetry	[122]
NPHP4	606966	AR	*nphp4*	MO	Pronephric cysts, curved body, hydrocephalus, pericardial edema	[120]
NPHP5	609254	AR	*iqcb1*	MO	Pronephric cysts, curved body, hydrocephalus, pericardial edema	[123]
NPHP6	610188	AR	*cep290*	MO	Pronephric cysts, curved body, hydrocephalus, retinitis pigmentosa, cerebellar defects	[124]
NPHP7	611498	AR	*glis2*	MO	Pronephric cysts, convergent extension defects, curved body, hydrocephalus, abnormal cardiac looping	[125]
NPHP9	613824	AR	*nek8*	MO	Pronephric cysts, developmental delay, curved body, abnormal cardiac looping	[126]
NPHP10	613615	AR	*sdccag8*	MO	Pronephric cysts, developmental delay, curved body, hydrocephalus	[127]
NPHP13	614377	AR	*wdr19*	MO	Pronephric cysts, hydrocephalus, microphthalmia, body curvature	[128]
NPHP15	614845	AR	*cep164*	MO	Ventral body axis curvature, abnormal heart looping, pronephric tubule cysts, hydrocephalus heart looping	[129]
SLNS9	616629	AR	*traf3ip1*	MO	Pronephric cysts, microphthalmia, retinitis pigmentosa	[53]
JBTS 1	213300	AR	*inpp5e*	MO, CRISPR	Left–right body axis asymmetry, microphthalmia and disruption of apicobasal polarity in morphants and pronephric cysts, pericardial effusion and body curvature in both morphants and mutants	[54,130]
JBTS 2	608091	AR	*tmem216*	MO	Pronephric cysts, body axis asymmetry, gastrulation defects	[131]
JBTS 3	608629	AR	*ahi1*	MO	Pronephric cysts, cardiac asymmetry, brain, eye and ear abnormalities	[132]
JBTS 6	610688	AR	*tmem67*	MO	Pronephric cysts, pronephric duct dilatation, notochord anomalies, abnormal eye formation	[133]
JBTS 7	611560	AR	*rpgrip1l*	MO	Gastrulation defects, shortened body axis, thin somites with broad lateral extensions, minor kinking of the notochord, underdeveloped anterior structures	[134]
JBTS 8	612291	AR	*arl13b*	Retroviral insertion	Pronephric cysts, curved body	[135]
JBTS 9	612285	AR	*cc2d2a*	ENU	Pronephric cysts, pericardial edema, curved body	[136]
JBTS 10	300804	XLR	*ofd1*	MO	Curved body, hydrocephalus, pericardial edema, randomized laterality of brain and heart	[137]
JBTS 11	613820	AD, AR	*ttc21b*	MO	Gastrulation defects, shortened body axis, kinking of the notochord, broadening of somites	[138]
BBS 1	209900	AR, DR	*bbs1*	MO	Pronephric cysts, convergent extension defects, curved body, hydrocephalus, abnormal heart looping	[125]
TSC 1	191100	AD	*tsc1a*	MO	Pronephric cysts, asymmetry defects, curved body	[139]
TSC 2	613254	AD	*tsc2*	ENU	Abnormal brain development, increased embryonic mortality, enlarged liver, abnormal cilia	[140]
Short-rib thoracic dysplasia with or without polydactyly	615630	AR	*ift172*	MO, Retroviral insertion	Ventral body-axis curvature, formation of renal cysts, cartilage defects with hypoplasia	[141,142]
	611263	AR	*ift80*	MO	Abnormal brain development, increased embryonic mortality, enlarged liver, abnormal cilia	[141]
	------------	AR	*tekt1*	MO	Ventral body-axis curvature, formation of renal cysts, cartilage defects with hypoplasia	[128]
Renal-hepatic ciliopathy	616217	AR	*dcdc2*	MO	Pronephric cysts, hydrocephalus, ventralized body axis, pericardial edema	[143]
Jeune thoracic dystrophy	616300	AR	*cep120*	MO	Abnormal body curvature, hydrocephalus, otolith defects, abnormal renal and craniofacial development	[144]
Ciliopathy *	------------	------------	*pik3r4*	MO	Pronephric cysts, hydrocephalus, curved body	[145]
**CAKUT**						
Papillorenal syndrome	616002	AD	*pax2a*	ENU	Abnormal pronephros development, defective tubular differentiation and patterning	[146]
DiGeorge syndrome	188400	AD	*crkl*, *aifm3*, *snap29*	MO, CRISPR	Major convolution defects, reduced length of pronephric tubules	[147]
Denys–Drash syndrome	194080	AD	*wt1a*	MO	Disruption of glomerular morphogenesis and differentiation	[148]
Renal cysts and diabetes syndrome	137920	AD	*hnf1ba,b*	MO, Retroviral insertion	Abnormal nephron segmentation, tubular dysfunction	[149]
Renal hypodysplasia	604994	AD	*six2*	MO	Altered renal morphology, dorsalization of the embryo	[150]
Renal hypodysplasia Bilateral renal agenesis *	112262	AD	*bmp4*	MO	Altered renal morphology, ventralization of the embryo	[150]
	------------	AD	*greb1l*	ENU, MO, CRISPR	Dilated tubules, deformed junction between proximal convoluted tubules and the neck, pronephric cysts, pericardial edema, early mortality	[151]
Classic bladder exstrophy	600057	XLR	*isl1*	MO	Abnormal urinary tract development	[152]
CAKUT1	612666	AD	*dstyk*	MO	Cloacal deformities, growth retardation, pericardial edema, small fins, abnormal jaw development	[153]

* For some recently reported genes, pathogenic mutations have been associated with a human renal phenotype but syndrome names and OMIM numbers have not been identified yet. AD, autosomal dominant; ADPKD, autosomal dominant polycystic kidney disease; ADTKD, autosomal dominant tubulo-interstitial kidney disease; AR, autosomal recessive; ARPKD, autosomal recessive polycystic kidney disease; BBS, Bardet–Biedl syndrome; CAKUT, congenital anomalies of the kidney and urinary tract; CRISPR, clustered regularly interspaced short palindromic repeats; ENU, N-ethyl-N-nitrosourea; FSGS, focal segmental glomerulosclerosis; JBTS, Joubert syndrome; MO, morpholino antisense oligonucleotides; NPHP, nephronophthisis; OMIM, Online Mendelian Inheritance in Man; RTA, renal tubular acidosis; SeSAME syndrome, seizures, sensorineural deafness, ataxia, mental retardation, electrolyte imbalance; SLNS, Senior–Loken syndrome; SRNS, steroid resistant nephrotic syndrome; TALENs, Transcription activator-like effector nucleases; TSC, tuberous sclerosis; XL, X-linked.
